# Reconfigurable THz Plasmonic Antenna Based on Few-Layer Graphene with High Radiation Efficiency

**DOI:** 10.3390/nano8080577

**Published:** 2018-07-28

**Authors:** Seyed Ehsan Hosseininejad, Mohammad Neshat, Reza Faraji-Dana, Max Lemme, Peter Haring Bolívar, Albert Cabellos-Aparicio, Eduard Alarcón, Sergi Abadal

**Affiliations:** 1 School of Electrical and Computer Engineering, University of Tehran, Tehran 14174, Iran; mneshat@ut.ac.ir (M.N.); reza@ut.ac.ir (R.F.-D.); 2 Department of Electrical Engineering, Yazd University, Yazd 89158, Iran; 3 Chair of Electronic Devices, RWTH Aachen University, 52074 Aachen, Germany; max.lemme@rwth-aachen.de; 4 AMO GmbH, 52074 Aachen, Germany; 5 Department of Electrical Engineering and Computer Science, University of Siegen, 57068 Siegen, Germany; peter.haring@uni-siegen.de; 6 NaNoNetworking Center in Catalonia (N3Cat), Universitat Politècnica de Catalunya, 08034 Barcelona, Spain; acabello@ac.upc.edu (A.C.-A.); eduard.alarcon@upc.edu (E.A.); abadal@ac.upc.edu (S.A.)

**Keywords:** graphene, plasmonics, terahertz band, tunable antenna, few-layer graphene, graphene stack

## Abstract

Graphene plasmonic antennas possess two significant features that render them appealing for short-range wireless communications, notably, inherent tunability and miniaturization due to the unique frequency dispersion of graphene and its support for surface plasmon waves in the terahertz band. In this letter, dipole-like antennas using few-layer graphene are proposed to achieve a better trade-off between miniaturization and radiation efficiency than current monolayer graphene antennas. The characteristics of few-layer graphene antennas are evaluated and then compared with those of antennas based on monolayer graphene and graphene stacks, which could also provide such improvements. To this end, first, the propagation properties of one-dimensional and two-dimensional plasmonic waveguides based on the aforementioned graphene structures are obtained by transfer matrix theory and finite-element simulation, respectively. Second, the antennas are investigated as three-dimensional structures using a full-wave solver. Results show that the highest radiation efficiency among the compared designs is achieved with the few-layer graphene, while the highest miniaturization is obtained with the even mode of the graphene stack antenna.

## 1. Introduction

Terahertz band communication is a key wireless technology that enables a plethora of applications at the macroscale and micro/nanoscale [1,2]. This technology requires the development of antennas capable of efficiently operating in this frequency range. For this purpose, graphene-based plasmonic antennas have been introduced as an attractive solution due to their unique tunability and miniaturization properties, as well as their Complementary Metal-Oxide-Semiconductor (CMOS) technology compatibility [3,4].

Different graphene plasmonic antenna designs have been investigated in the literature, often highlighting the abovementioned significant advantages over conventional antennas, which are given by the unique frequency dispersion of graphene and its ability to support surface plasmon polaritons (SPPs) in the terahertz band [4,5,6,7,8]. These designs generally consider a monolayer graphene structure, but the radiation efficiency of such antennas is low. Other configurations such as few-layer graphene or certain graphene stacks would theoretically lead to higher radiation efficiency thanks to their higher conductivity and mode coupling, respectively. However, few-layer graphene has been considered for plasmonic waveguides with high confinement [9] and for antennas as an auxiliary element [10], but not as the radiating element of plasmonic antennas; whereas graphene stacks have been introduced in [11,12] seeking self-biased reconfigurability, not better radiation efficiency.

In this paper, we investigate the use of few-layer graphene in plasmonic antennas with the aim of obtaining a graceful balance between efficiency and miniaturization. Particularly, we evaluate the radiation efficiency, miniaturization, and impedance characteristics of dipole-like antennas based on few-layer graphene for a wide set of parameter values and compare them to those of a monolayer counterpart. For a more complete comparison, we also consider graphene stacks as they can also provide high radiation efficiency and miniaturization thanks to their mode coupling and field confinement potential.

**Figure 1:**
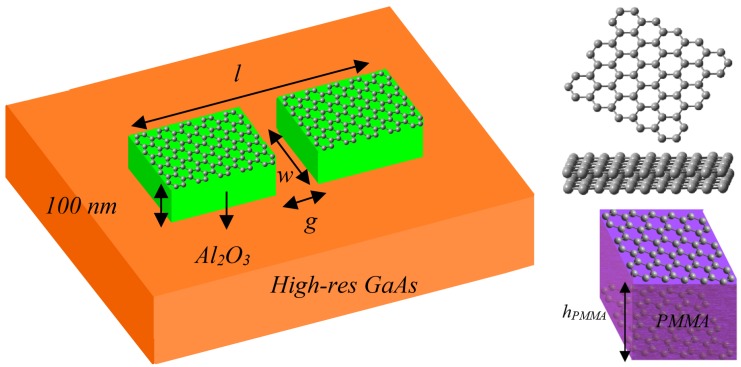
Configuration of dipole antenna by considering monolayer graphene (MLG, **top**), few-layer graphene (FLG, **middle**), and a graphene stack (GS, **bottom**).

The antenna under study is basically a dipole of total length *l* and width *w*, as depicted in Figure 1. Dipole arms are composed of a graphene structure, which can be either monolayer graphene, few-layer graphene, or a graphene stack, deposited on top of a thin dielectric layer –Aluminum Oxide (Al_2_O_3_) in our case. In the graphene stack case, a polymethylmethacrylate (PMMA) layer is used to act as a dielectric between the two graphene sheets. The substrate is considered high resistivity Gallium Arsenide (GaAs). To bring the design closer to experimental realization, we assume the dipole to be fed at its center gap of length *g* with a terahertz photomixer that acts as a photoconductive source [3]. It is worth mentioning that all the antennas considered in this work are compatible with widespread and even commercially available growth, transfer, and patterning methods for graphene [3,12,13].

The remainder of the paper is as follows. In Section 2, the investigation of the plasmonic modes supported by the different graphene waveguides is performed. This serves as a starting point for the design of graphene antenna structures, which are detailed and analyzed in Section 3. The paper is concluded in Section 4.

## 2. One-Dimensional and Two-Dimensional Plasmonic Waveguides Based on Graphene

In order to design and analyze an efficient plasmonic antenna, the investigation of the surface plasmon mode supported by its guided-wave structure is a primary step. Simplification of two-dimensional structures to one-dimensional structures is a reasonable starting point for their analysis. Therefore, consider three slab waveguides composed of monolayer graphene (MLG), few-layer graphene (FLG), and graphene stack (GS) as shown in Figure 1. The resulting one-dimensional structures are thus GaAs-Al_2_O_3_-MLG-Air, GaAs-Al_2_O_3_-FLG-Air, and GaAs-Al_2_O_3_-GS-Air.

Although there is not a definitive consensus on the definition of FLG, most works consider it to be graphene sheets with no more than five layers [9,13,14]. The optical conductivity of FLG is $N\sigma_{G}$ [13], where $\sigma_{G}$ is the surface conductivity of MLG calculated by the Kubo formula [15] and *N* is the number of layers. The reason for defining FLG for N≤5 is that this conductivity relation does not hold for more than five layers as interactions between adjacent layers cannot be ignored anymore and the conductivity approaches the values of bulk graphite [13]. Throughout the paper, we consider two FLG instances: FLG2 for bi-layer graphene and FLG5 for the limit case of FLG with five layers. Intuitively, the higher conductivity of FLG should eventually lead to lower losses and, thus, higher efficiency.

In the case of GS, two graphene monolayers are separated by PMMA dielectric with height $h_{PMMA}$. This value is chosen so that monolayers are far enough to disregard coupling through quantum effects [16]. Dielectric constants $\varepsilon_{r}$ of GaAs, Al_2_O_3_, and PMMA are assumed 12.9, 9, and 2.4, respectively. The layer thickness of both Al_2_O_3_ and PMMA are assumed 100 nm, well within fabrication limits [12], whereas the excitation frequency is 3 THz. Intuitively, antennas based on GS could provide higher efficiency through mode coupling and higher miniaturization due to their field confinement potential as sought in waveguide designs [9].

The transverse magnetic (TM) modes supported by the SPP waves for the waveguides are studied using the formulations of transfer matrix theory provided in our previous work [17]. It is worth mentioning that there are two possible modes for GS structure, namely, the even mode, in which the normal electric fields is even symmetric; and the odd mode, in which the symmetry is odd.

**Figure 2:**
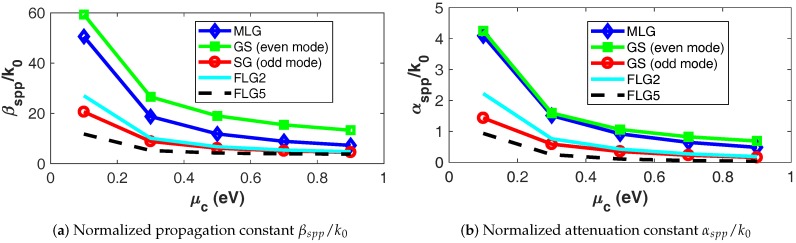
Normalized propagation and attenuation constant of the structures as a function of the chemical potential at 3 THz.

Based on previous experiments and compatible with existing growth, encapsulation, and biasing techniques [3,12,14], we assume the temperature, relaxation time, chemical potential of graphene to be 300 K, 0.6 ps, and 0.1–0.9 eV, respectively. With these figures, the normalized propagation constant $\beta_{spp}/k_{0}$ and normalized attenuation constant $\alpha_{spp}/k_{0}$ of the evaluated structures are shown in Figure 2.

These results regarding the behavior of plasmonic guided-wave structures can be employed to reason about the performance of graphene antennas as follows. In order to compare the miniaturization potential of the different antennas, the $\beta_{spp}/k_{0}$–$\mu_{c}$ chart shown in Figure 2a is a suitable measure because the resonant length is inversely proportional to the effective index of the propagated mode. We thus observe that the even mode of GS structure has the highest confinement for the $\mu_{c}$ values here considered, i.e., $\beta_{spp}/k_{0}=$ 59.3–13.3, as expected; whereas the structure based on FLG with five layers has the lowest compactness among all structures, but with a suitable confinement factor nonetheless, i.e., $\beta_{spp}/k_{0}=$ 11.8–3.8. FLG2 lies between MLG and FLG5.

On the other hand, a suitable qualitative measure of superiority of the structures from the perspective of radiation efficiency is the $\alpha_{spp}/k_{0}$–$\mu_{c}$ chart shown in Figure 2b. The reason is that the radiation efficiency has an inverse relation with the losses of the propagated mode. It is thus observed that the antenna based on FLG with five layers potentially provides the best radiation efficiency among the compared structures, as expected. Again, FLG2 shows a compromise between the efficiency of FLG5 and the miniaturization of MLG.

**Figure 3:**
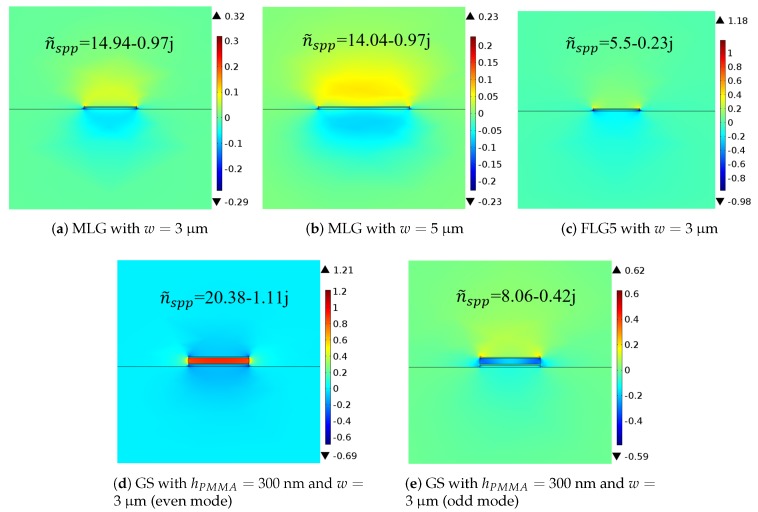
Normal electric fields and effective refractive indices of different structures with *μ_c_* = 0.5 eV at 3 THz.

Let us now return to the two-dimensional guided-wave structure. We used the finite element method of the COMSOL field solver to perform the numerical calculations, including normal electric fields and effective refractive indices ${\tilde{n}}_{spp}$ of the structures as shown in Figure 3. It is observed that the results of two-dimensional structures are similar to those of one-dimensional structures: FLG5 shows the lowest Im$\left\{{\tilde{n}}_{spp}\right\}$ denoting lowest losses, whereas GS has the highest Re$\left\{{\tilde{n}}_{spp}\right\}$ denoting highest confinement. The dependence with the width of the strip becomes clear: narrower strips are characterized by larger effective refractive index due to the higher confinement of moving charges. As discussed subsequently, this affects the antenna performance.

## 3. THz Plasmonic Antenna Based on MLG, FLG and GS

The antennas made of three kinds of guided-wave structures (MLG, FLG, and GS) are investigated as three-dimensional structures by the full-wave solver of the software CST Studio Suite.

**Figure 4:**
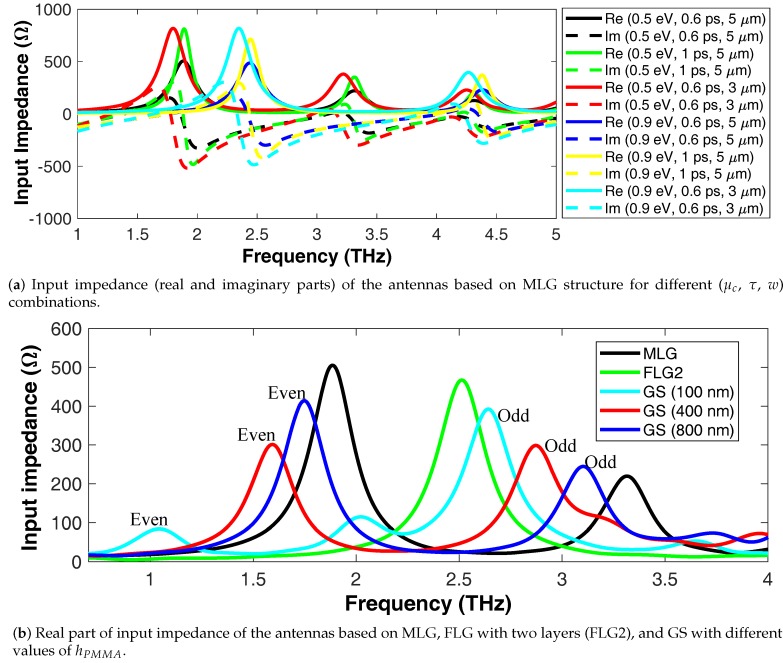
Input impedance characterization of the proposed antenna structure.

Assuming structural parameters of *l* = 12 μm and *w* = 3–5 μm, the real and imaginary parts of the input impedance of a MLG-based antenna are as shown in Figure 4a. Different values for the parameters τ and $\mu_{c}$, related to the graphene sheets, are considered. It is observed that the first resonance of the antenna, i.e., the imaginary part of the impedance is zero, has a low input impedance and it may be thus seen as a short-circuit resonance. The second resonance where the real and imaginary parts of input impedance are large can be interpreted as an open-circuit resonance. Contrary to in microwave antennas, the open-circuit resonance may be desired because it reduces the return loss when connecting the plasmonic antenna to THz sources that generally have high impedance. Considering *l* = 12 μm, *w* = 3 μm, g = 2 μm, τ = 0.6 ps, and $\mu_{c}=$ 0.5 eV, the open-circuit resonance ($f_{r}$, $\lambda_{r}$ when $l=l_{r}$) occurs at 1.8 THz, which is lower than the $l_{r}=\lambda_{spp}=\frac{\lambda_{0}}{n_{spp}}$ frequency predicted by the transmission line model because the dipole gap has non-negligible capacitance in this geometry.

In order to study the antennas based on FLG structure and compare them with antenna based on MLG structure, we present radiation efficiency $e_{r}$, miniaturization factor $\lambda_{r}/L$, and input impedance $Z_{in}$ in Table 1 for two values of chemical potential (0.5 and 0.9 eV), relaxation time (0.6 and 1 ps), and strip width (3 and 5 μm). For the sake of brevity, we only present results for FLG with five layers. The results contained therein can be explained using the conclusions drawn from the guided-wave structures in Section 2 as follows.

In both structures, changing chemical potentials from 0.5 to 0.9 eV causes the $\beta_{spp}$ to decrease, therefore leading to a higher resonant frequency. Consequently, $e_{r}$ is greater because the antenna becomes electrically larger at resonance, while miniaturization factor $\lambda_{r}/L$ is reduced. On the other hand, this change causes $\alpha_{spp}$ to decrease; resulting in the higher radiation efficiency because of a reduction of the ohmic loss of the antenna. In addition, implementing graphene with higher τ, which introduces lower loss, and also increasing *w*, which provides a larger electrically antenna, both result in a higher radiation efficiency.

Higher confinement by the use of a narrower strip or lower Fermi level increases the value of the input impedance. Moreover, higher τ (lower losses) causes a sharper resonance, resulting in larger input impedance in the open-circuit case.

Results also confirm that the FLG5 has higher radiation efficiency and lower miniaturization than MLG-based antennas. This is because the guided-wave structure based on FLG5 provides a mode propagating with a lower loss and a lower confinement compared to MLG structure. Since the conductivity of FLG5 starts to be comparable to that of bulk graphite, we do not expect that adding more layers would result in significant improvements. In any case, efficiency would increase slightly and miniaturization would drop.

Let us now compare the antenna based on FLG (only FLG2 with two layers for the sake of brevity), GS with different $h_{PMMA}$ values, and MLG with l= 12 μm, w= 5 μm, $\mu_{c}=$ 0.5 eV, and τ= 0.6 ps to further study miniaturization. Input impedance of the antennas is illustrated in Figure 4b. The results can be explained by the effective indices of plasmonic modes as follows. The refractive indices of the structures based on MLG is 14.04, on FLG2 is 8.44, whereas on GS (odd and even modes) are 8.08 and 29.44 for $h_{PMMA}=$ 100 nm, 7.31 and 18.43 for $h_{PMMA}=$ 400 nm, and 6.43 and 16.15 for $h_{PMMA}=$ 800 nm at 3 THz. In light of these results, the most miniaturized antenna can be achieved with the even mode of GS structure. It should be mentioned that the results of GS antennas with large and small amounts of $h_{PMMA}$ is similar to MLG-based and FLG-based antennas, respectively. Generally, the results show that there is a tradeoff between miniaturization and radiation efficiency and the antenna based on FLG presents a suitable compromise, which can be further modulated by chosing the number of layers.

## 4. Conclusions

This paper has proposed few-layer graphene as a promising material for the realization of THz reconfigurable high radiation efficiency plasmonic antenna. The choice is driven by its higher conductivity with respect to monolayer graphene. Through analytical and numerical methods, we confirmed that few-layer graphene antennas offer high efficiency at the cost of losing some miniaturization potential. It has been also observed that the choice of number of layers allows to navigate the efficiency–miniaturization tradeoff. A comprehensive comparison to graphene stacks, which are a valid alternative with potential for low losses and high confinement, has further confirmed that the highest radiation efficiency is achieved by the few-layer graphene structure, while the highest miniaturization is supported by the even mode of graphene stack structure.

## Figures and Tables

**Table 1:** Radiation efficiency ($e_{r}$), resonant frequency ($f_{r}$), miniaturization factor ($\lambda_{r}/L$), and input impedance ($Z_{in}$) of MLG antennas and FLG antennas with five layers (FLG5) for different parameters.

Antenna	$\mu_{c}$ (eV)	τ (ps)	*w* (μm)	$e_{r}$	$f_{r}$ (THz)	$\lambda_{r}/L$	$Z_{\mathrm{in}}$ (Ω)
	0.5	0.6	3	3%	1.8	13.9	818
	0.5	0.6	5	5%	1.9	13.2	500
MLG	0.5	1	5	8%	1.9	13.2	800
	0.9	0.6	3	11%	2.35	10.6	817
	0.9	0.6	5	19%	2.45	10.2	485
	0.9	1	5	28%	2.45	10.2	712
	0.5	0.6	3	50%	3.8	6.5	450
FLG5	0.9	0.6	5	69%	4.4	6	325
	0.9	1	5	78%	4.4	6	365

## References

[1] Akyildiz I.F., Jornet J.M., Han C. (2014). Terahertz band: Next frontier for wireless communications. Phys. Commun..

[2] Abadal S., Alarcón E., Cabellos-Aparicio A., Lemme M., Nemirovsky M. (2013). Graphene-enabled Wireless Communication for Massive Multicore Architectures. IEEE Commun. Mag..

[3] Cabellos A., Llatser I., Alarcón E., Hsu A., Palacios T. (2015). Use of THz Photoconductive Sources to Characterize Tunable Graphene RF Plasmonic Antennas. IEEE Trans. Nanotechnol..

[4] Tamagnone M., Perruisseau-Carrier J. (2014). Predicting Input Impedance and Efficiency of Graphene Reconfigurable Dipoles Using a Simple Circuit Model. IEEE Antennas Wirel. Propag. Lett..

[5] Vakil A., Engheta N. (2011). Transformation optics using graphene. Science.

[6] Luo X., Qiu T., Lu W., Ni Z. (2013). Plasmons in graphene: Recent progress and applications. Mater. Sci. Eng. R Rep..

[7] Hosseininejad S.E., Komjani N. (2016). Comparative analysis of graphene-integrated slab waveguides for terahertz plasmonics. Photonics Nanostruct.-Fundam. Appl..

[8] Hosseininejad S.E., Alarcón E., Komjani N., Abadal S., Lemme M.C., Haring Bolívar P., Cabellos-Aparicio A. (2017). Study of hybrid and pure plasmonic terahertz antennas based on graphene guided-wave structures. Nano Commun. Netw..

[9] Zhou X., Zhang T., Chen L., Hong W., Li X. (2014). A graphene-based hybrid plasmonic waveguide with ultra-deep subwavelength confinement. J. Light. Technol..

[10] Hosseininejad S.E., Komjani N. (2016). Waveguide-fed tunable terahertz antenna based on hybrid graphene-metal structure. IEEE Trans. Antennas Propag..

[11] Tamagnone M., Gómez-Díaz J.S., Mosig J.R., Perruisseau-Carrier J. (2012). Reconfigurable terahertz plasmonic antenna concept using a graphene stack. Appl. Phys. Lett..

[12] Gómez-Díaz J.S., Moldovan C., Capdevila S., Romeu J., Bernard L., Magrez A., Ionescu A., Perruisseau-Carrier J. (2015). Self-biased reconfigurable graphene stacks for terahertz plasmonics. Nat. Commun..

[13] Casiraghi C., Hartschuh A., Lidorikis E., Qian H., Harutyunyan H., Gokus T., Novoselov K.S., Ferrari A.C. (2007). Rayleigh imaging of graphene and graphene layers. Nano Lett..

[14] Gan C.H. (2013). Analysis of surface plasmon excitation at terahertz frequencies with highly-doped graphene sheets via attenuated total reflection. App. Phys. Lett..

[15] Gusynin V.P., Sharapov S.G., Carbotte J.P. (2006). Magneto-optical conductivity in graphene. J. Phys. Condens. Matter.

[16] Velasco J., Lee Y., Jing L., Liu G., Bao W., Lau C.N. (2012). Quantum transport in double-gated graphene devices. Solid State Commun..

[17] Hosseininejad S.E., Komjani N., Noghani M.T. (2015). A Comparison of Graphene and Noble Metals as Conductors for Plasmonic One-Dimensional Waveguides. IEEE Trans. Nanotechnol..

